# Networks of Indirect Contact Promote Spread of an Environmentally Transmitted Pathogen in a Highly Social Species

**DOI:** 10.1002/ece3.73719

**Published:** 2026-05-27

**Authors:** Jack R. Leitch, Neil R. Jordan, John M. Drake, Michael A. Cant, Kathleen A. Alexander

**Affiliations:** ^1^ Department of Fish and Wildlife Conservation Virginia Tech Blacksburg Virginia USA; ^2^ CARACAL, Centre for Conservation of African Resources: Animals, Communities, and Land Use Kasane Botswana; ^3^ Centre for Ecosystem Science, University of New South Wales, School of Biological, Earth and Environmental Sciences Sydney New South Wales Australia; ^4^ Taronga Wildlife Reproductive Centre Taronga Conservation Society Australia Dubbo New South Wales Australia; ^5^ Botswana Predator Conservation Maun Botswana; ^6^ Odum School of Ecology University of Georgia Athens Georgia USA; ^7^ Center for the Ecology of Infectious Diseases University of Georgia Athens Georgia USA; ^8^ University of Exeter Penryn UK

**Keywords:** banded mongoose, contact network, environmental transmission, *Mycobacterium mungi*, scent marking, temporal network

## Abstract

In group‐living species, social behaviors, including non‐contact territorial defense, may decrease the risk of pathogen transmission and spread by reducing contact among conspecifics, yet these strategies may paradoxically increase opportunities for transmission in some host‐pathogen systems. We explore how social behaviors shape direct and indirect pathogen transmission of the emerging *Mycobacterium tuberculosis* complex (MTBC) pathogen *M. mungi* in banded mongooses ( *Mungos mungo* ). Using empirical behavioral data from a banded mongoose population in Uganda, we constructed social network models that incorporate observed intra‐group contacts and simulated inter‐group scent marking scenarios. These models show that indirect transmission can play a pivotal role, particularly when per‐contact transmissibility is low. Simulations predicted increased population‐level spread via socially directed, environmentally mediated pathways, while population‐level invasion potential remained relatively unchanged. These results underscore the need to incorporate all transmission modes when modeling disease spread in social networks. We further emphasize the importance of considering the temporal dynamics of indirect transmission, including the persistence of both olfactory secretion and infecting pathogens deposited in the environment. For mixed transmission systems, fully accounting for both direct and indirect transmission mechanisms may be essential for characterizing disease spread, even when direct transmission pathways dominate. Our findings suggest that pathogens that exploit socially directed environmental transmission pathways may gain an evolutionary advantage, circumventing social barriers thought to have evolved to constrain pathogen spread.

## Graphical abstract

Using behavioral observations and network simulations, we evaluate direct and indirect transmission of the MTBC pathogen *M. mungi* in banded mongooses. Incorporating intergroup scent marking reveals that environmentally mediated, socially directed pathways can promote spread at low transmissibility, elevating population prevalence even when direct contact is primary. These results argue for routinely including indirect routes and their temporal dynamics in social‐network models and control strategies.

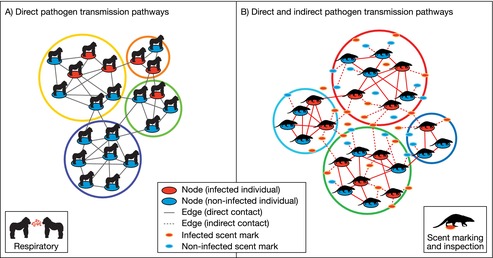

## 1 Introduction

For highly social species, group living offers many advantages, but it also carries costs (Krause and Ruxton 2002). Among these, group living generally increases both individual‐level pathogen infection risk and potential for population‐level spread. Within groups, higher rates of contact among conspecifics translate to additional opportunities for direct pathogen transmission, and greater density may intensify exposure to indirectly transmitted pathogens (Altizer et al. 2003). The effects of social behaviors on infection risk are highly context dependent. For example, allogrooming may reduce ectoparasite burdens, enhancing resistance or tolerance to pathogens (Ezenwa et al. 2016; Loehle 1995), while simultaneously increasing exposure to directly transmitted microparasites through close physical contact (Altizer et al. 2003). Non‐contact territorial defense, typically achieved through olfactory, visual, or auditory communication, reduces direct contact between groups of conspecifics, further inhibiting spread of pathogens across the population (Loehle 1995; White et al. 2020). However, pathogens may exploit these social behaviors and communication mechanisms to promote their transmission, compromising the effectiveness of these strategies in reducing disease risk.

**FIGURE 1:**
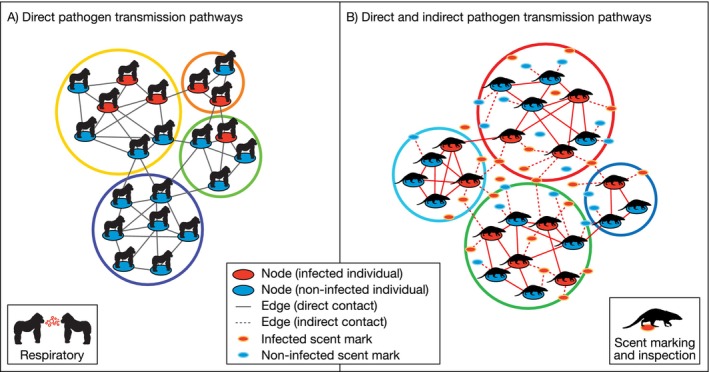
Conceptual illustration of how social networks and pathogen transmission may differ between pathogens transmitted directly and those that are transmitted both directly and indirectly. (A) In many host‐pathogen systems, transmission is influenced by social contact network structures determined by patterns of direct physical contact between susceptible and infected conspecifics. (B) Scent marking behavior in banded mongooses creates additional opportunities for transmission of *M. mungi*, circumventing social barriers to pathogen spread.

By facilitating or obstructing pathogen transmission, social behaviors shape the social network of transmission opportunities, thereby determining individual infection risk, pathogen invasion potential, and patterns of spread across the population. Social behaviors that drive pathogen deposition in the environment and subsequent exposure can result in directed environmental transmission that is more than incidental. Incorporating these socially‐directed indirect transmission opportunities into social network models could substantially alter the topology of these networks and may therefore be critical for understanding and predicting pathogen spread (Figure 1).

Many studies have identified the significant influence of host social networks on pathogen transmission dynamics in wildlife when directly transmitted pathogens move and spread among conspecifics (Böhm et al. 2009; Drewe 2010; Hamede et al. 2009). Indirect pathogen transmission has also been investigated, though studies have typically focused on explicitly spatial aspects of transmission and the probability of individuals encountering pathogen in the environment based on patterns of shared space use (White et al. 2020; Dougherty et al. 2018; Fofana and Hurford 2017; White et al. 2018). In contrast to network models that focus exclusively on direct transmission, integrating indirect transmission opportunities strengthens existing connections between individuals and establishes links between those that are not already connected (Shahzamal et al. 2019). This results in greater potential for pathogen invasion, higher peak and endemic prevalences, and increased host fatality rates for lethal pathogens (Almberg et al. 2011). Indirect transmission may even play a predominant role, enabling pathogens with very low infectivity to spread throughout an entire population (Richardson and Gorochowski 2015). Additionally, the relative influence of individuals in pathogen spread may vary substantially when considering indirect transmission as compared with direct transmission alone (Egan et al. 2023).

In the case of *Mycobacterium mungi*, a novel *Mycobacterium tuberculosis* complex (MTBC) pathogen that infects banded mongooses ( *Mungos mungo* ) in northern Botswana, both direct and indirect transmission routes are facilitated by social behaviors and communication (Alexander et al. 2016). Banded mongooses are small fossorial mammals that live in social groups (troops) consisting of 8–70 individuals (Cant 2000). Infected mongooses shed *M. mungi* in anal secretions and urine, with invasion occurring through breaks in the nasal planum and/or skin of susceptible conspecifics. As a result, direct pathogen transmission may occur when mongooses mark or groom each other (Alexander et al. 2015). *M. mungi* may also be transmitted indirectly through environmental scent marking. In banded mongooses, both sexes scent mark, investigate, and over‐mark (Jordan et al. 2010), depositing various eliminative (urine and feces) and glandular (anal and cheek) secretions (Jordan et al. 2010; Müller and Manser 2007) that can harbor viable mycobacteria (Alexander et al. 2015). Scent marking occurs throughout the home range, not exclusively at territorial boundaries, suggesting its importance in both intra‐group communication and territorial inter‐group communication (Jordan et al. 2010), with scent marking sites sometimes shared between neighboring groups (Jordan et al. 2011).

Olfactory communication behavior, such as scent marking, involves the environmental deposition of substances (e.g., urine, feces, anal secretions) that convey signals and information to conspecifics attracted to the secretions (Gorman and Trowbridge 1989; Jordan et al. 2013). Across a number of host‐pathogen systems, these excretions serve as potential routes for environmental pathogen transmission. For instance, *Trypanosoma cruzi*, a vector‐borne zoonosis, is found in the anal gland secretions of infected common opossums ( *Didelphis marsupialis* ), with environmental transmission occurring through ingestion (Deane et al. 1984). Opossums in the *Didelphis* genus exhibit complex scent marking behavior akin to banded mongooses (Kimble 1997), potentially increasing pathogen transmission risk for conspecifics and other spillover hosts. Similarly, squirrelpox virus (SQPV, family *Poxviridae*) transmission from gray squirrels ( *Sciurus carolinensis* ) to red squirrels ( *Sciurus vulgaris* ) may occur via surface contamination with anal gland secretions, suggesting that scent‐marking behavior in gray squirrels could influence spillover to red squirrels (Bruemmer et al. 2010). In white‐tailed deer ( *Odocoileus virginianus* ), scraping behavior used for communication during the breeding season involves deposition of secretions and bodily fluids, with subsequent investigation by conspecifics, possibly leading to the transmission of prions that cause chronic wasting disease (CWD) (Egan et al. 2023). Lastly, Iberian wolves ( *Canis lupus* ), like other canids, excrete feces in particular locations for communication between and within social groups, which may facilitate canine parvovirus‐2 (CPV) transmission through socially driven placement and inspection of contaminated feces used in olfactory communication (Millán et al. 2016).

For network models to effectively capture the dynamics of socially directed transmission in a particular host‐pathogen system, they must reflect the specific behaviors involved and how these behaviors translate to potential for pathogen transmission. Models incorporating both direct and indirect transmission should address varying transmissibility between direct and environmental routes, possibly accounting for differences in interaction strength within pairs of individuals or between individuals and the environment. Importantly, direct transmission requires simultaneous social presence (“same place, same time”), while indirect transmission only necessitates interaction with the same location without temporal concurrence (“same place, different time”) (Shahzamal et al. 2019; Egan et al. 2023; Silk et al. 2018). A pathogen's transmissibility in the environment might change over time following deposition—it may decay rapidly (Shahzamal et al. 2019), remain highly persistent (Egan et al. 2023), or experience a delay between deposition and infectivity (Leu et al. 2010). Models should account for the probability of susceptible hosts encountering the pathogen, which may also change over time. This becomes particularly important for pathogen transmission facilitated by environmental scent marking, where the signal itself degrades in the environment (Richardson and Gorochowski 2015). Temporal network models may offer a more accurate representation of the characteristics of such systems by explicitly accounting for these time‐related aspects (Silk et al. 2018).

In this study, we examine how social behaviors with potential for direct and indirect transmission influence invasion and spread of a pathogen through a host's social network. We construct a representative social network using detailed observation data from an uninfected population of banded mongooses. We translate this observed social network into a temporal contact network model and examine mathematical properties of the network that indicate potential for pathogen spread. We simulate pathogen spread across the networks with varying pathogen transmissibilities, illustrating the relative effects of direct and indirect transmission.

Recognizing the potential impact of inter‐group scent marking on pathogen spread, we construct additional contact network models by supplementing the observed network with synthetic inter‐group scent marking contacts, generated stochastically to represent plausible but unobserved overmarking scenarios. These augmented networks are exploratory, designed to examine how inter‐group marking behavior could influence pathogen spread if it occurs as hypothesized. Again, we examine the mathematical properties of these networks and simulate pathogen spreading to explore the potential impacts of scent marking on pathogen transmission.

## 2 Methods

### 2.1 Study Site and Behavioral Data Collection

Behavioral data used in this study were collected from December 2005 to November 2007 as part of previously published field studies of scent‐marking and social behavior in banded mongooses on and around Mweya Peninsula, Queen Elizabeth National Park, Uganda (0°11′20″S, 29°15′40″E; for details of the study area, see Cant (Cant 2000)). These studies included seven (in 2006) or eight (in 2007) wild groups of habituated, individually identifiable banded mongooses. Details of the original scent‐marking observations and associated field methods are provided elsewhere (Jordan et al. 2010; Jordan et al. 2011).

No new animal handling was conducted for this study. The original fieldwork was conducted under approval from the Ethical Committee of the Department of Zoology at the University of Cambridge and under permits from the Uganda Wildlife Authority and the Uganda National Council for Science and Technology, in accordance with ASAB/ABS guidelines for the treatment of animals in behavioral research and teaching (*Animal Behaviour* 2006).

### 2.2 Individual Interactions

For the present analysis, we extracted individual‐level behaviors with potential for direct or indirect pathogen transmission from the previously collected behavioral observation dataset. These included scent deposition, scent inspection, allomarking, anogenital inspection, and allogrooming. Behavioral observations were recorded using all‐occurrence sampling, including the identities of the actor and the recipient mongoose and whether the behavior was reciprocated. Coordinates of observed scent‐marking sites were recorded using a hand‐held GPS device. During scent marking, feces and urine were deposited in token amounts, with urination involving a stereotyped stamping of the hind legs, while anal marks and cheek marks involved rubbing either the anal pouch or the side of the “face” along objects or the ground (Jordan et al. 2010). Scent marks can be overmarked and/or inspected through sniffing or licking of the deposit. Scent marks were excluded from the data if no further mongoose contacts were observed after secretions were deposited. These excluded events may nonetheless represent pathogen deposition in the environment with potential for unobserved subsequent contact, and their exclusion likely contributes to a conservative estimate of indirect transmission.

Allogrooming was recorded when a mongoose nibbled or inspected the fur of another mongoose repeatedly, potentially coming into contact with infected secretions. In some cases of mutual allogrooming, the individual initiating the behavior was unseen and could not be recorded. When three or more animals were grooming together in a huddle, all interactions were recorded as dyads (one record per dyad), regardless of the duration of grooming or the number of times each animal switched back and forth between partners. This representation may modestly inflate direct‐contact counts for grooming huddles involving three or more individuals. However, given that indirect contacts outnumber direct contacts by more than an order of magnitude in our network, this is unlikely to substantively affect our findings. A separate grooming bout was considered to have started if the animals resumed grooming after a pause (during which there was no grooming at all) of more than 1 min. Allomarking was recorded when one individual scent‐marked the body of a conspecific, allowing potential pathogen invasion through injuries or further allogrooming behaviors. Anogenital inspection was recorded when one individual sniffed the anogenital region of another mongoose, exposing the investigating mongoose to potentially infected anogenital secretions.

### 2.3 Observed Temporal Contact Network

Using the mongoose behavioral observation data, we constructed a multiplex directed temporal contact network model (hereafter, “observed network”, “Obs”). A contact network model allows us to represent individual heterogeneity of contacts as well as contact patterns arising from social structure, both of which are known to have potentially significant effects on pathogen spread (Lloyd‐Smith et al. 2005; Salathé and Jones 2010). The nodes (representing individual banded mongooses) in our network remain fixed while the edges (representing direct and indirect contacts with potential for pathogen transmission) vary over time. Use of temporal contact networks retains the fundamental ordering of contacts (Bansal et al. 2010), which can be particularly important because the dynamics of contacts and pathogen spread in this host‐pathogen system occur on similar time scales (Pastor‐Satorras et al. 2015).

Each node in our observed network represents a mongoose in the observed population (N=364), forming the node set $V=\left\{v_{1},\ldots ,v_{N}\right\}$. The node set was fixed with respect to time, despite births, deaths, and other potential factors resulting in individual mongooses being observed for only a subset of the total observation period.

Observed contacts between individuals were aggregated into two independent sets by mode of transmission, direct (D) or indirect (I), with the type of behavior indicating direction of potential pathogen transmission. For each observed behavior, including scent marking, the type of behavior determines the direction of potential pathogen transmission. We incorporated this directionality into the model through directed edges. For allomarking, potential for pathogen transmission was directed from the marking individual to the individual being marked, with the reverse contact also included when allomarking was reciprocal. For instances of anogenital inspection, potential transmission was directed from the individual being inspected to the inspecting individual. For allogrooming, potential transmission was directed from the individual being groomed to the individual performing the grooming, again with reverse contact included when allogrooming was reciprocal.

**FIGURE 2:**
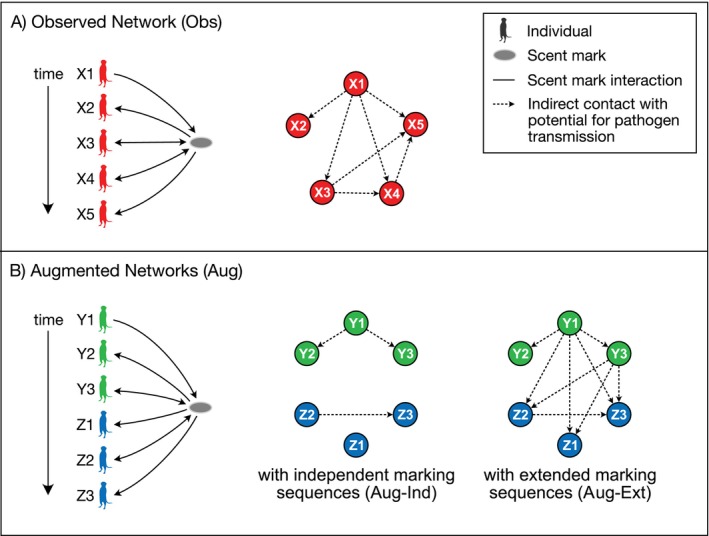
Construction of temporal contact networks from behavioral observation data. (Obs, observed network; Aug‐Ind, augmented network with independent overmarking sequences; Aug‐Ext, augmented network with extended overmarking sequences). Sequences of scent marking behaviors for each marking site (left) included interactions in which individuals potentially deposit pathogen at the site or may be exposed to previously deposited pathogen, based on the sequence and types of interactions. Arrows indicate direction of potential pathogen movement between individuals and the marking site. Color of individuals and individual identifier indicate group affiliation. (A) For the observed network (Obs), each sequence of intra‐group marking interactions (left) was translated into indirect contacts between members of the group (right). (B) For the augmented networks, each sequence of marking interactions (left) was translated into two sets of indirect contacts (right), one in which indirect contacts were only included if between members of the same group (Aug‐Ind) and another which included indirect contacts both within and between groups (Aug‐Ext).

For each sequence of interactions with a marking site, we counted potential indirect transmission directed from each individual depositing a mark to all who subsequently interacted with the same site on the same day (see Figure 2A). Formally, for a sequence of interactions with a marking site ${\left(m_{i},b_{i}\right)}_{i=1}^{n}$, where $m_{i}$ is an individual mongoose and $b_{i}$ is a marking behavior, we counted potential indirect transmission from $m_{i}$ to $m_{j}$ for all i,j such that $b_{i}$ represents deposition of a mark, i<j, and $m_{i}\neq m_{j}$.

We aggregated observed contacts with potential for direct and indirect pathogen transmission independently into two sets of weighted adjacency matrices $W_{t}^{\left(D\right)}$ and $W_{t}^{\left(I\right)}$, with entries $W_{t,\mathrm{ij}}^{\left(D\right)}$ and $W_{t,\mathrm{ij}}^{\left(I\right)}$ representing the number of contacts with potential for direct (D) and indirect (I) transmission respectively, directed from i to j, at time step t. Contacts were aggregated to weekly snapshots for time steps t=1,…,T=97.

The node set V, along with the two weighted adjacency matrices, define the observed multiplex temporal network $O=\left(V,W_{t}^{\left(D\right)},W_{t}^{\left(I\right)}\right)$. Separating contacts with potential for direct and indirect pathogen transmission into two edge layers enables examination of pathogen spreading under varying combinations of direct ($\beta_{D}$) and indirect ($\beta_{I}$) transmissibility.

In our temporal network, potential pathogen transmission occurs along causal paths (Holme 2005), which can be conceptualized as conduits in time and across individuals, through which pathogens may spread and persist (Holme and Saramäki 2012). To measure the potential for pathogen spreading, we computed the number of unique causal paths in the network, using software package *pathpy*, version 2.2.0 (Hackl et al. 2021). We also computed the reachability ratio $\rho_{i}$ for each node i, defined as the fraction of all nodes that are in the set of influence of i (the set of nodes that are reachable from i via causal paths). In this context, the reachability ratio determines an upper bound on prevalence $\rho_{i}+1/N$ when node i is the index case in a completely susceptible population, regardless of disease dynamics. Under susceptible‐infected (SI) dynamics with transmissibilities $\beta_{D}=\beta_{I}=1$ (guaranteed transmission for each contact from an infected individual to a susceptible individual), final prevalence is determined by this upper bound, equal to $\rho_{i}+1/364$.

### 2.4 Simulation of Pathogen Spreading

Next, we simulated stochastic spreading of a hypothetical pathogen with susceptible‐infected (SI) dynamics across the observed network. For each simulation run, we infected a single individual with the hypothetical pathogen (with all others susceptible) and allowed the pathogen to spread stochastically across O, with direct ($\beta_{D}$) and indirect ($\beta_{I}$) transmissibility fixed across all edges and time steps. Infection status of nodes at each time step t was tracked using vectors ${\left(\chi_{t}\right)}_{t=0}^{T}$ of length N, where $\chi_{t,i}=1$ if node i is infected after time step t and 0 otherwise. Assuming independence of potential transmission across contacts, the probability of a susceptible node *j* ($\chi_{t-1,j}=0$) becoming infected ($\chi_{t,j}=1$) is given by

$$P\left(\chi_{t,j}=1|\;\chi_{t-1}\right)=1-{\left(1-\beta_{D}\right)}^{\chi_{t-1}W_{t,j}^{\left(D\right)}}{\left(1-\beta_{I}\right)}^{\chi_{t-1}W_{t,j}^{\left(I\right)}}$$

Without recovery under SI disease dynamics, prevalence for the overall simulation period is then equal to the prevalence after the final time step, $\frac{1}{N}\sum_{j}\chi_{T,j}$. Varying transmissibilities across all pairs $\left(\beta_{D},\beta_{I}\right)$ for $\beta_{D}$ and $\beta_{I}$ in $\left\{0,0.05,0.1,\ldots ,0.95,1\right\}$, we repeated the simulation ten times with each node as the index case, for a total of 1,605,240 independent runs.

We calculated the basic reproduction number ($R_{0}$) according to its definition for each pair $\left(\beta_{D},\beta_{I}\right)$, using the record of transmission events that occurred during each simulation run. The mean number of secondary cases arising from the index case was calculated across the ten runs of the simulation for each index case, with the mean of these means subsequently yielding $R_{0}$. Formally, for a given parameter set $\left(\beta_{D},\beta_{I}\right)$, let $s_{\mathrm{ik}}$ denote the number of secondary cases arising from index case i in simulation run k. Then $R_{0}$ was calculated as $R_{0}=\frac{1}{N}\sum_{i}\left[\frac{1}{10}\sum_{k}s_{\mathrm{ik}}\right]$, where N=364 is the number of individuals in the population.

We assessed relative influence of direct and indirect pathogen transmissibility for determining final prevalence and $R_{0}$ in our simulations using standardized coefficients from fitted linear regression models. For each outcome, we fit an additive model with $\beta_{I}$ and $\beta_{D}$ as predictors. The ratio of the absolute standardized coefficients, $\left|{\hat{\gamma }}_{I}\right|/\left|{\hat{\gamma }}_{D}\right|,$ indicates the relative influence of indirect transmissibility compared with direct transmissibility in predicting the output variable (prevalence or $R_{0}$).

### 2.5 Augmented Temporal Contact Networks

The scent marking observations used in constructing the observed network O include intra‐group events only. However, marking sites may be further overmarked by other groups, which may enable inter‐group transmission of a pathogen like *M. mungi*. While inter‐group overmarking has been documented in banded mongooses (Jordan et al. 2011), the frequency and structure of these interactions were not captured in our observational data. To explore the potential effects of these additional unobserved scent marking interactions, we constructed sets of 100 temporal contact networks ${\left(E_{k},S_{k},U_{k}\right)}_{k=1}^{100}$ stochastically augmented with synthetic inter‐group marking contacts, designed to isolate different aspects of inter‐group marking. In augmented‐extended networks $E_{k}$ (“Aug‐Ext”), synthetic marking sequences extend the observed marking event so that any pathogen deposited through the observed marking has potential for transmission to members of an overmarking neighboring group (see Figure 2B). In augmented‐independent networks $S_{k}$ (“Aug‐Ind”), the synthetic marking sequences are added as separate events that do not extend the original marking sequences, meaning that pathogen deposited through the observed marking cannot be transmitted to the neighboring group. In augmented‐uniform networks $U_{k}$ (“Aug‐Unif”), the synthetic marking sequences extend the observed events as in $E_{k}$, but individual participation and marking behaviors are assigned uniformly at random rather than weighted by observed behavioral patterns. Comparison of $E_{k}$ and $S_{k}$ therefore isolates the contribution of inter‐group transmission pathways, while comparison of $E_{k}$ and $U_{k}$ isolates the contribution of behaviorally structured individual participation.

To identify potential inter‐group interactions for the augmented networks, for each marking sequence in the observational data for group *X*, ${M_{X}=\left(x_{i},b_{i}\right)}_{i=1}^{n}$, we calculated the probability $\pi_{X,Z}$ that each other group (*Z*) would encounter and overmark the site, extending the sequence of marking contacts. This is the joint probability of the marking site being within the portion of group *X*'s home range that overlaps with group *Z*'s home range ($p_{s}$) and the probability that group *Z* will encounter and overmark a site within its home range ($p_{e}$), $\pi_{X,Z}=p_{s}p_{e}$. Banded mongooses typically deposit scent marks throughout the home range rather than preferentially in areas that border or overlap with other groups' home ranges (Jordan et al. 2010). Without knowing the specific location of the marking site, we calculated $p_{s}$ as the fraction of group *X*'s home range that overlapped with group *Z*'s home range, based on 95% kernel home range overlap. We assumed that a group would encounter and overmark a site within its home range with fixed probability $p_{e}=0.1$. We then determined stochastically, based on $\pi_{X,Z}$, whether each group encountered and overmarked group *X*'s marking sequence. If, by chance, multiple groups were selected to encounter and overmark, one of the groups was chosen uniformly at random from those selected.

To extend the marking sequence $M_{X}$, we generated a synthetic sequence of marking behaviors $M_{Y}={\left(y_{j},b_{j}\right)}_{j=1}^{m}$ for group *Y*, with length m sampled from the discrete distribution of observed marking sequence lengths. The mongoose performing the marking behavior $y_{j}$ was selected from the membership of group *Y* on the date of the marking event, sampled by sex according to frequency of marking behavior by males and females in the observational data. The marking behavior $b_{j}$ was sampled from the distribution of frequencies of observed marking behavior types. By sampling from the empirical distributions, we attempted to preserve potential effects of sex and behavior on pathogen spread.

For each of the augmented networks $E_{k}$, $S_{k}$, and $U_{k}$, we calculated the number of unique causal paths in the network and reachability ratio for each node, and then simulated stochastic spreading of a hypothetical pathogen with SI dynamics, all as described above for the observed network. Stochastic spreading simulations were performed using a pathogen with fixed transmissibilities $\beta_{D}=0.1$ and $\beta_{I}=0.01$. These values were selected to yield plausible spread across the network while reflecting the expectation that per‐contact transmissibility is lower for environmentally mediated indirect transmission than for direct transmission. We ran the simulation ten times for each node as the index case, a total of 36,400 independent runs per augmented network. We calculated $R_{0}$ for each augmented network, using the fixed values of $\beta_{D}$ and $\beta_{I}$, as described above for the observed network.

## 3 Results

### 3.1 Potential for Pathogen Invasion and Spreading

**FIGURE 3:**
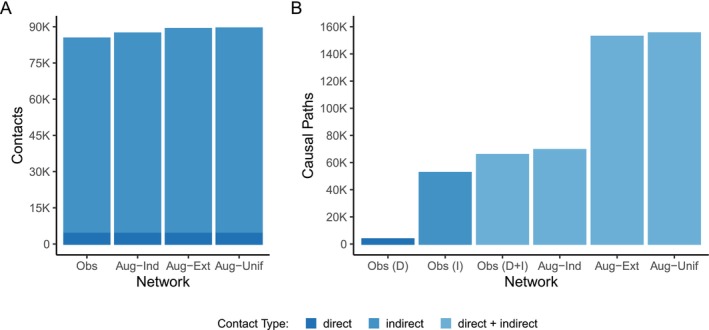
Contacts and causal paths for the observed and augmented networks. (Obs, observed network; Aug‐Ind, augmented network with independent overmarking sequences; Aug‐Ext, augmented network with extended overmarking sequences; Aug‐Unif, augmented network with unstructured individual participation) (A) Interactions with marking sites resulted in many more indirect contacts than direct contacts in the observed network. Indirect contacts increased slightly for the augmented networks with independent (Aug‐Ind), extended (Aug‐Ext), and unstructured individual participation (Aug‐Unif) marking site interactions, and Aug‐Ext and Aug‐Unif had similar contact counts. (B) The number of causal paths was roughly proportional to the number of contacts for all networks except the augmented network with extended marking sequences (Aug‐Ext) and the augmented network with unstructured individual participation (Aug‐Unif), owing to the presence of inter‐group synthetic marking contacts.

Overall, we observed 1894 instances of allogrooming (including reciprocation), 703 instances of allomarking (including reciprocation), and 2424 instances of anogenital inspection over the observation period, for a total of 5021 observed contacts with potential for direct pathogen transmission. The observational data also contained 24,944 instances of intra‐group scent marking interactions, which translated to 80,224 effective contacts with potential for indirect transmission. When standardized by the number of individuals active in the population each week, mongooses were involved in a mean of 0.32 (SD 0.30) contacts with potential for direct transmission per individual per week and 5.59 (SD 8.85) contacts with potential for indirect transmission per individual per week (median indirect contact rate = 2.93, reflecting right‐skewed weeks of intensive marking activity). Following the addition of synthetic scent marking interactions representing plausible overmarking scenarios, the augmented networks with extended overmarking sequences (Aug‐Ext) included an average of 89,192 (range 87,459–91,957) effective contacts, compared with 87,325 (range 86,352–89,109) for augmented networks with independent overmarking sequences (Aug‐Ind) and 89,426 (range 87,567–92,167) for augmented networks with unstructured individual participation (Aug‐Unif) (Figure 3A).

The observed network was found to contain 65,779 unique causal paths; 3663 when considering only direct contacts (D); 52,532 when considering only indirect contacts (I). The augmented networks with extended overmarking sequences (Aug‐Ext) had on average 152,806 (range 123,095–195,543) causal paths, compared with an average of 69,394 (range 66,667–72,335) for the augmented networks with independent overmarking sequences (Aug‐Ind) and 155,338 (range 123,522–208,415) for the augmented networks with unstructured individual participation (Aug‐Unif) (Figure 3B). The ratio of causal paths to number of contacts was 0.77 for the observed network, mean 1.71 for Aug‐Ext, mean 1.78 for Aug‐Ind, and mean 1.74 for Aug‐Unif.

**FIGURE 4:**
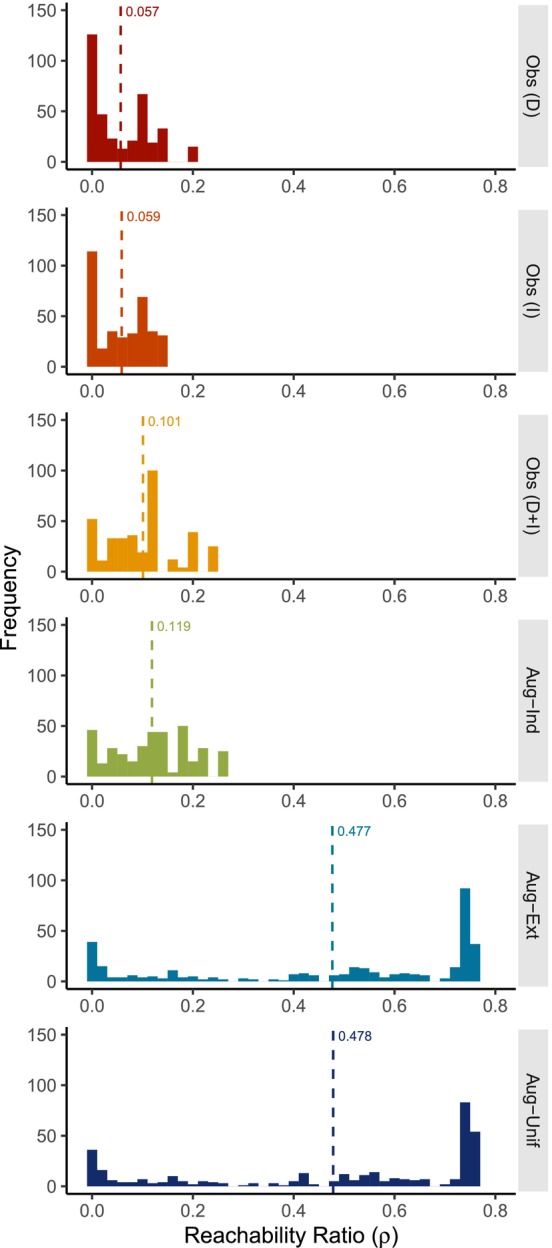
Frequency of reachability ratios for the observed and augmented networks. (Obs, observed network; Aug‐Ind, augmented network with independent overmarking sequences; Aug‐Ext, augmented network with extended overmarking sequences; Aug‐Unif, augmented network with unstructured individual participation). Vertical dashed lines indicate mean reachability ratios across all individuals in the study population. Reachability ratios when considering only direct (D) or only indirect (I) observed contacts were similar. Mean reachability ratio increased when both direct and indirect observed contacts were considered together (D + I). Synthetic contacts added in the augmented‐independent network (Aug‐Ind) increased the mean reachability ratio slightly. Extended marking interactions substantially increased the mean reachability ratio in the augmented‐extended network (Aug‐Ext). Reachability was similar between Aug‐Ext and the augmented network with unstructured individual participation (Aug‐Unif).

Reachability ratios (Figure 4) were similar when considering observed direct contacts only (D) (mean 0.057, range 0.00–0.20) versus indirect contacts only (I) (mean 0.059, range 0.00–0.13). When both direct and indirect contacts were considered (D + I), reachability ratios (mean 0.10, range 0.00–0.25) were greater than for either modality alone, and had a lower frequency of individuals with zero reachability (D + I: 45, D: 88, I: 109). Reachability ratios for the augmented networks with extended overmarking sequences (Aug‐Ext) (mean 0.48, range 0.00–0.76) were far greater than for the observed network. Reachability ratios for the augmented networks with independent overmarking sequences (Aug‐Ind) (mean 0.12, range 0.00–0.25) were only slightly increased relative to the observed network. Reachability ratios for the augmented networks with unstructured individual participation (Aug‐Unif) were similar to Aug‐Ext (mean 0.48, range 0.00–0.76).

### 3.2 Simulated Pathogen Spread

**FIGURE 5:**
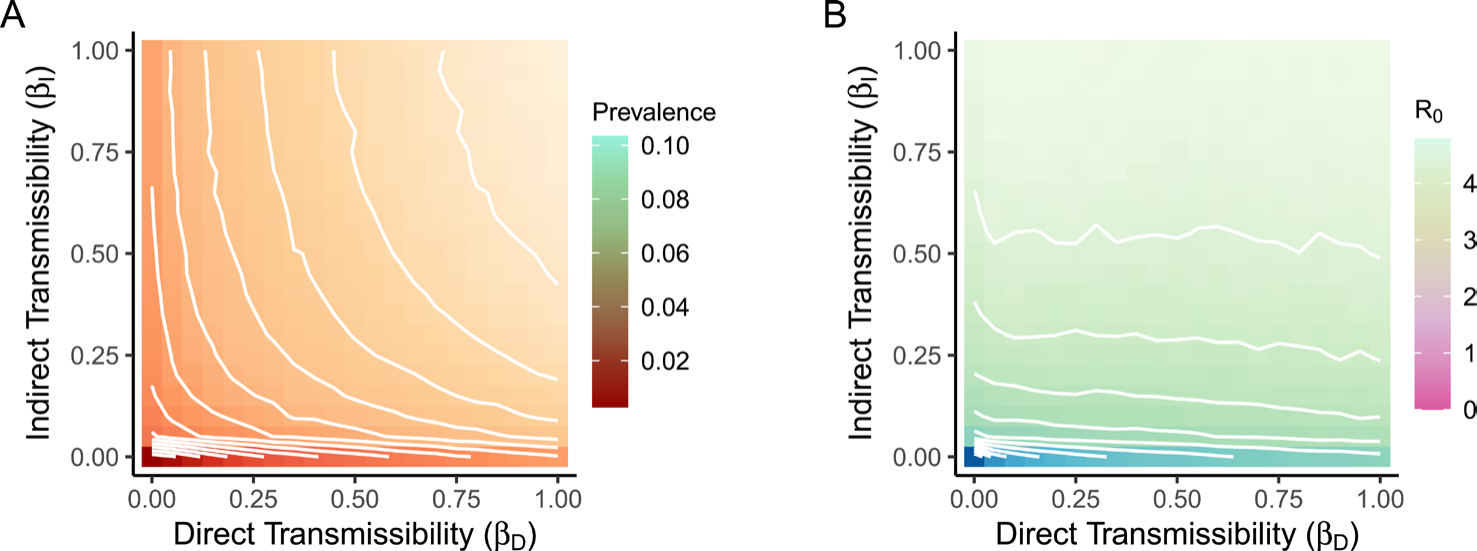
Overall prevalence and basic reproduction number ($R_{0}$) for spreading simulations on the observed network, by transmissibility of the hypothetical pathogen. White lines indicate contours, combinations of direct and indirect transmissibility values that result in the same prevalence or $R_{0}$ value. (A) Prevalence increased rapidly for small increases in indirect transmissibility near zero. (B) $R_{0}$ increases rapidly for small increases in indirect transmissibility near zero.

In simulations of pathogen spread on the observed network, mean prevalence ranged from 0.003 ($\beta_{D}=0.00$, $\beta_{I}=0.00$) to 0.103 ($\beta_{D}=1.00$, $\beta_{I}=1.00$) (means over 10 stochastic simulations per index case per parameter set) over all combinations of direct ($\beta_{D}$) and indirect ($\beta_{I}$) transmissibility values (Figure 5A). At the low end of this range, zero transmissibility limits infection to the index case only, resulting in a prevalence value of 0.003 (1 out of 364 mongooses infected). Prevalence increased uniformly with respect to both direct and indirect transmissibility. For low transmissibility values ($0.00\leq \beta_{D}\leq 0.15$, $0.00\leq \beta_{I}\leq 0.15$), indirect transmissibility was 2.37 times as important as direct transmissibility in predicting prevalence based on standardized regression coefficients ($\left|\beta_{I}^{*}\right|/\left|\beta_{D}^{*}\right|=2.37;$ multiple linear regression was statistically significant, $R^{2}=0.94$, $F\left(2,14\right)=103.05$, p<0.001). In comparison, across the entire range of transmissibility values ($0.00\leq \beta_{D}\leq 1.00$, $0.00\leq \beta_{I}\leq 1.00$), indirect transmissibility was 0.91 times as important as direct transmissibility in predicting prevalence ($R^{2}=0.94$, $F\left(2,439\right)=3,361.36$, p<0.001).

For the observed network, the mean basic reproduction number ($R_{0}$) measured from simulation ranged from 0.0 ($\beta_{D}=0.00$, $\beta_{I}=0.00$) to 4.8 ($\beta_{D}=0.08$, $\beta_{I}=1.00$) (means over 10 stochastic simulations per index case per parameter set; Figure 5B). For low transmissibility values ($0.00\leq \beta_{D}\leq 0.15$, $0.00\leq \beta_{I}\leq 0.15$), indirect transmissibility was 2.28 times as important as direct transmissibility in predicting $R_{0}$ (multiple linear regression was statistically significant, $R^{2}=0.93$, $F\left(2,14\right)=94.95$, p<0.001). Across the entire range of transmissibility values ($0.00\leq \beta_{D}\leq 1.00$, $0.00\leq \beta_{I}\leq 1.00$), indirect transmissibility was 1.58 times as important as direct transmissibility in predicting $R_{0}$ ($R^{2}=0.91$, $F\left(2,439\right)=2,162.08$, p<0.001).

**FIGURE A1:**
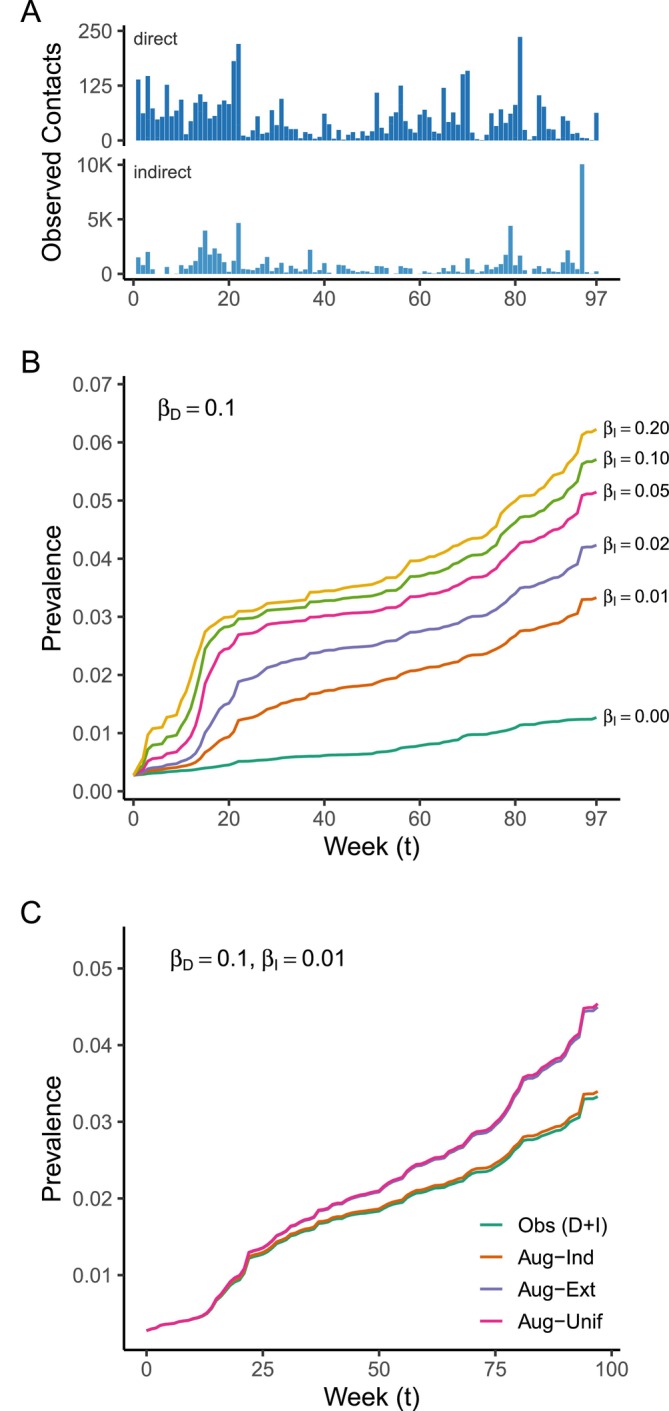
Observed contacts and prevalence in simulations. (Obs, observed network; Aug‐Ind, augmented network with independent overmarking sequences; Aug‐Ext, augmented network with extended overmarking sequences; Aug‐Unif, augmented network with unstructured individual participation). (A) Observed contacts per week for the observation period. (B) Prevalence for representative indirect transmissibility values ($\beta_{I}$) with fixed direct transmissibility ($\beta_{D}=0.1$). (C) Prevalence for the observed network, augmented‐independent network, augmented‐extended network, and augmented network with unstructured individual participation, for fixed direct and indirect transmissibility values $\beta_{D}=0.1$ and $\beta_{I}=0.01$. Shaded areas indicate the range of prevalence at each time period over all stochastic simulations; in some cases, these ranges are narrow due to low variance across runs.

For simulations of pathogen spread under fixed transmissibilities $\beta_{D}=0.10$ and $\beta_{I}=0.01$ (Appendix Figure A1), prevalence was greater for the augmented networks with extended overmarking sequences (mean 0.045, range 0.040–0.051) and the augmented networks with unstructured individual participation (mean 0.045, range 0.041–0.053) than for the augmented networks with independent overmarking sequences (mean 0.034, range 0.033–0.035) and for the observed network (0.033). The basic reproduction number ($R_{0}$) was also greater for the augmented networks with extended overmarking sequences (mean 2.20, range 2.15–2.27) and the augmented networks with unstructured individual participation (mean 2.21, range 2.14–2.27) than for those with independent overmarking sequences (mean 2.16, range 2.09–2.22) and for the observed network (2.15).

## 4 Discussion

Although certain social behaviors and non‐contact territorial defense strategies are generally considered to decrease pathogen exposure potential, this study suggests that they may paradoxically promote pathogen transmission and spread in certain systems such as *M. mungi* within banded mongoose populations. Within social groups, the combination of direct and indirect transmission opportunities results in a greater number of causal paths and higher mean reachability than either direct or indirect transmission opportunities alone, indicating an increased potential for spread. Our simulations reveal that the combination of direct and indirect contacts, particularly those arising from environmental scent marking behaviors, substantially increases the basic reproduction number ($R_{0}$) and overall prevalence. Notably, in our simulations, indirect transmission was found to be more than twice as important as direct transmission in predicting invasion potential and spread for low, and arguably more biologically plausible, transmissibility values. These findings suggest the possibility that pathogens may evolve transmission mechanisms that circumvent social strategies conventionally thought to reduce pathogen transmission.

Under our simulated inter‐group overmarking scenarios, inclusion of synthetic inter‐group environmental scent marking contacts in the social network considerably increases potential for spread across the population. In our network models, the number of causal paths more than doubled, and mean reachability increased by nearly a factor of four. Under modest assumptions for direct and indirect pathogen transmissibility, simulations affirm that this increase in potential for spread results in substantially higher prevalence when compared with scenarios without these synthetic contacts, or when the addition of synthetic contacts does not result in additional causal paths between groups. Interestingly, $R_{0}$ increases only modestly, suggesting that while inter‐group scent marking offers greater opportunity for population spread, it does not necessarily increase the potential for pathogen invasion. The contrast between substantially higher prevalence and only modestly increased $R_{0}$ is consistent with inter‐group transmission seeding new intra‐group outbreaks that amplify within groups but do not fundamentally alter invasion dynamics. This is further supported by the comparison between Aug‐Ext and Aug‐Ind, which share similar numbers of contacts but differ in whether inter‐group causal paths exist. The higher prevalence in Aug‐Ext relative to Aug‐Ind suggests that the bridging of groups through extended marking sequences, and the resulting potential for intra‐group amplification following inter‐group seeding, drives the increase in population‐level spread. The comparable results between Aug‐Ext and Aug‐Unif further indicate that this amplification does not depend on the specific individuals involved or the types of marking behaviors performed, but rather on the sequential structure of scent marking interactions at shared sites that generates inter‐group transmission pathways. This observation is consistent with mathematical predictions that invasion in networks with pronounced community structure often proceeds more readily through dense intra‐community links than through more limited inter‐community links (Salathé and Jones 2010). It also suggests that social structure and behaviors remain effective in mitigating pathogen invasion in this host‐pathogen system. Increasing $R_{0}$ would require greater pathogen transmissibility, potentially leading to infection rates considerable enough to tip the system from endemicity to extirpation.

Indirect transmission driven by social behaviors can significantly amplify pathogen spread as compared with indirect transmission that is not socially directed. This study attributes the prominent contribution of indirect contacts to transmission largely to the specific behaviors of banded mongooses during scent marking interactions. Rapid, successive interactions with each marking site result in a dramatic increase in contacts with potential for indirect transmission (Figure 3). Consequently, substantial transmission may occur via indirect exposure, even when per‐contact indirect transmissibility is low. With scent marking, individuals preferentially interact with marks, markedly increasing potential for pathogen deposition and exposure. As a form of non‐contact territorial defense, indirect transmission via scent marks can connect individuals and social groups with limited spatial overlap, unlike environmental transmission occurring solely through shared space use (e.g., random fecal deposition), which is density dependent.

Considering the substantial impact of socially directed indirect transmission on pathogen spread and the prevalence of associated behaviors across species, it seems crucial to account for possible indirect routes. Both spatial and social modes may operate independently or jointly within a host‐pathogen system, underscoring the need to understand how these distinct modes contribute to the spread within a particular system. It is therefore imperative to examine not only proximity or location, but also the behaviors occurring in these locations which can facilitate pathogen deposition and exposure, the effectiveness of the communication mechanism in attracting attention, and the ability of the pathogen to remain infectious in the environment. It is equally important to account for how the contributions of these factors change over time. A pathogen may remain infectious and transmissible in the environment long after the communication mechanism fades, which would shift the transmission mode from socially directed to purely spatial. This may be seen, for example, in the case of scraping communication and transmission of prions that cause chronic wasting disease in white‐tailed deer, where prions may remain infectious in the environment for years, long after scrapes have faded (Egan et al. 2023).

These findings underscore the importance of incorporating behaviorally driven indirect contacts alongside direct contacts in social‐network models of infection. In socially structured host populations, different transmission modes may contribute unequally and interact in ways that alter both invasion and spread. Models that omit socially driven indirect transmission may therefore underestimate pathogen spread and obscure the mechanisms that connect transmission within and between groups.

Acknowledging the inherent temporal dynamics of indirect pathogen transmission, it is imperative to incorporate aspects of time in social network models. This necessitates the use of temporal networks and temporally explicit metrics, with pathogen transmission occurring solely through time‐respecting causal paths (Holme and Saramäki 2012). Further work is needed to define temporally explicit metrics, understand their mathematical characteristics, and outline best practices for their use in understanding and predicting pathogen transmission dynamics. For example, in our study, we estimated $R_{0}$ through simulation, but it may be possible to more rigorously estimate potential for invasion using a more generalized and temporally explicit epidemic threshold (Leitch et al. 2019).

Because the social networks analyzed here were constructed from observational data for a population of banded mongooses not infected with *M. mungi*, susceptible‐infected (SI) disease dynamics were required for our simulations. Introduction of a “removed” class, for example, using susceptible‐infected‐removed (SIR) dynamics, would likely more accurately represent this host‐pathogen system, as would incorporation of individual heterogeneity in pathogen shedding and host susceptibility. Similarly, our model does not explicitly couple demographic processes with infection dynamics. The contact data reflect which individuals were present in the population each week, and individuals entering the population during the observation period are implicitly treated as immunologically naïve. However, infection‐driven mortality or dispersal could dampen spread and trigger social disruption such as group fission and fusion, known to occur in infected mongoose groups (Fairbanks et al. 2014), which could substantially alter the network. Additionally, our simulations assume homogeneous per‐contact transmissibility across all individuals, which does not account for variation likely to occur in natural host–pathogen systems, such as superspreading individuals or pathogen resistance and acquired immunity that can dampen spread within groups or populations. Addressing these complexities would require a generative network with probabilistic contacts parametrized from observational data, which would permit simulation with a variety of transmission probabilities as well as the influence of group and demographic dynamics.

While transmissibility per effective contact for *M. mungi* in banded mongooses has not yet been estimated, the representative values used in this analysis ($\beta_{D}=0.1,\beta_{I}=0.01$) yield plausible spread across the social network. We also assumed that scent marks and pathogen do not persist in the environment beyond the day of deposition, which may be quite conservative given that, across multiple host‐pathogen systems, scent marks may remain detectable with viable pathogen for much longer (Fine et al. 2011; Martinez et al. 2019). Across many mammalian species, scent marks have been found to persist to the extent that they are investigated by conspecifics for at least several weeks following deposition (10–20 days for anal gland secretion of dwarf mongooses, *Helogale rufula* (Rasa 1973); at least 30 days for anal‐gland pastings of brown hyenas (Gorman and Mills 1984); 23 days for urine of wolves, *Canis lupus* (Peters and Mech 1975); 40 days for urine of guinea pigs, *Cavia porcellus* (Wellington et al. 1981); at least 40 days for scent marks of leopards, *Panthera pardus* (Rafiq et al. 2020)). Thus, while there is limited information with which to parametrize our model, the parameters we have chosen may lead to significant underestimation of the effects of indirect contact on population spread.

As we have seen here, accounting for indirect pathogen transmission via the environment can be critical to understanding key features of pathogen spread in host‐pathogen systems, and the effects of indirect transmission may be large even when transmission is primarily through direct contact. Particularly in group living species, dense within‐group contacts readily amplify potential for intra‐group direct pathogen transmission, while indirect transmission across groups via environmentally mediated pathways enables the pathogen to access new groups where further within‐group amplification may occur. This suggests increased evolutionary fitness for pathogens that can exploit these indirect transmission mechanisms—the very mechanisms that are believed to have evolved to reduce risk of infection and spread across populations.

## Author Contributions

**Jack R. Leitch:** conceptualization (equal), data curation (equal), formal analysis (lead), investigation (lead), methodology (lead), software (lead), visualization (lead), writing – original draft (lead), writing – review and editing (equal). **Neil R. Jordan:** data curation (equal), writing – review and editing (equal). **John M. Drake:** writing – review and editing (equal). **Michael A. Cant:** writing – review and editing (equal). **Kathleen A. Alexander:** conceptualization (equal), funding acquisition (lead), methodology (equal), supervision (lead), writing – original draft (equal), writing – review and editing (equal).

## Funding

This work was supported by the National Science Foundation, 1518663, 1918770, 2009717.

## Conflicts of Interest

Kathleen Alexander is the board president of CARACAL. CARACAL was a sub‐recipient on a grant that funded this work (NSF award #2009717).

## Data Availability

The code and data that support the findings of this study are openly available in Dryad at https://doi.org/10.5061/dryad.f1vhhmh8n.
